# Determinants for Congenital Clubfoot in Ethiopia: A Case‐Control Study at Black Lion Specialized Hospital

**DOI:** 10.1002/hsr2.72328

**Published:** 2026-04-14

**Authors:** Seid Mohammed Abdu, Birhanu Ayana, Girma Seyoum

**Affiliations:** ^1^ Department of Medical Anatomy, School of Biomedical Science, College of Medicine and Health Sciences Wollo University Dessie Amhara Region Ethiopia; ^2^ Department of Orthopedic, School of Medicine, College of Health Sciences Addis Ababa University Addis Ababa Ethiopia; ^3^ Department of Medical Anatomy, School of Medicine, College of Health Sciences Addis Ababa University Addis Ababa Ethiopia

**Keywords:** case‐control, clubfoot, congenital clubfoot, determinants, Ethiopia, risk factor

## Abstract

**Background:**

Clubfoot is a frequently occurring congenital deformity and continues to be overlooked public health concern, especially among children under‐fives year old in low and middle‐income countries, where approximately 90% of cases occur in these developing world. Despite this significant burden, no observational studies in Ethiopia have examined the factors influencing clubfoot deformities. To bridge this gap, our study aimed to investigate the determinants of congenital clubfoot among children attended the black lion hospital.

**Material and Methods:**

An institution‐based, unmatched case‐control study was carried out at Black Lion Specialized Hospital. A total of 284 participants (96 cases and 188 controls) were enrolled to identify potential risk factors for congenital clubfoot. SPSS 25 was used for analysis, and terms like frequency, percentage, and logistic regression were used for data analysis.

**Result:**

In this study of 284 participants, several factors were significantly associated with clubfoot included lack of formal education (AOR = 8.29), chronic illness (AOR = 2.57), smoking exposure (AOR = 4.83), alcohol history (AOR = 5.39), male (AOR = 2.08), and family history of clubfoot (AOR = 12.78).

**Conclusion:**

This research revealed key determinants of congenital clubfoot, including low education, male, family history, exposure to smoking, chronic illnesses, and alcohol use. The finding point out the importance of raising awareness and empowering pregnant women to identify and manage potential clubfoot determinants, and nutritional support during pregnancy.

**Abbreviations**
HMIS: Health management information system
MRN: medical record number

## 1 Introduction

Clubfoot is a condition characterized by a complicated misalignment of the foot, impacting both the soft and bony structures [1]. If untreated the deformity progresses into Neglected Clubfoot, where ongoing walking exacerbates the deformity, leading to pain and reduced mobility due to further stretching of soft and bony tissues [2].

It is major, yet neglected, public health problems particularly affecting young children in economically disadvantaged countries. In 2017 alone, ~675,061 children under five globally were affected by clubfoot, with Southern Asia bearing the highest burden, followed by Eastern Asia and Eastern Africa [3, 4]. Furthermore, each year, ~200,000 babies worldwide are born with clubfoot, with nearly 90% of these cases occurring in developing countries [5]. The incidence vary globally, ranging from 1 to 3 per 1000 live births globally [6, 7, 8]. Similarly, studies from Africa, report the birth prevalence of 1.11 per 1000 live births [9].

The underlying causes of clubfoot cases are not yet fully understood. However, idiopathic clubfoot, is likely influenced by multiple factors [10, 11]. A genetic component is almost certainly involved [12], while environmental influences, seasonal variations, and in utero positioning have also been proposed as potential contributors. However, these factors have not been consistently confirmed [10].

Although maternal and environmental factors have been implicated in the development of clubfoot, the evidence remains limited, with reported associations often inconsistent and inadequately investigated. Factors described in the literature include young maternal age [13, 14, 15, 16, 17], diabetic mother [15], low educational level [14, 15, 16], cigarettes smoker [14, 15, 16], family history [15], and joint exposure of smoking and family history [16, 18, 19] paternal smoking [15, 16], maternal body mass index [15], amniocentesis, selective serotonin reuptake inhibitor exposure [15], male sex [15, 20, 21, 22], null parity [15], winter months [16].

Despite the substantial burden of clubfoot as one of the most frequently observed congenital deformities in low‐ and middle‐income countries (LMICs), including Africa, studies examining its potential risk factors remain extremely limited, particularly in developing regions such as Ethiopia. Most available literature from Africa focuses on treatment outcomes, implementation of the Ponseti method, or descriptive epidemiology, with virtually no analytical case–control studies investigating etiological factors. Therefore, this study represents one of the first case–control investigations in Ethiopia and aims to assess risk factors associated with congenital clubfoot, providing critical insights that may inform early detection strategies and preventive interventions.

## 2 Materials And Methods

### 2.1 Study Design

Hospital based unmatched case‐control study was conducted among patient with congenital club foots and controls without congenital club foot in the study period at Black Lion Specialized Hospital, Addis Ababa, Ethiopia, 2020. It was conducted for three consecutive months from September 20–November 30, 2021. The study was conducted in the Orthopedics Ward of the Pediatric Orthopedics Clinic at Black Lion Specialized Hospital in Addis Ababa, Ethiopia. This department offers extensive musculoskeletal care, advanced training programs, and research opportunities. Staffed by consultant orthopedic surgeons and resident trainees, it serves as a leading referral center in Ethiopia. The hospital receives patients from across the country, managing a wide range of cases, including fractures, trauma emergencies, and congenital musculoskeletal conditions such as clubfoot.

### 2.2 Population

#### 2.2.1 Source Population

The source population consisted of all children under 5 years of age who attended the pediatric orthopedic clinic at Black Lion Specialized Hospital during the study period. Black Lion Specialized Hospital is a referral center, and while cases and controls were not entirely drawn from the same catchment area, many children came from areas near the hospital. Therefore, the study population may not fully represent all children in the community.

#### 2.2.2 Study Population

All pediatric patients, who presented in Black Lion Specialized Hospital for both cases and controls during the study periods.

### 2.3 Eligibility Criteria

#### 2.3.1 Inclusion Criteria

##### 2.3.1.1 Cases

‐ This study included children younger than 5 years who were diagnosed with congenital clubfoot and received treatment at the Pediatric Orthopedic Clinic within the Orthopedics Department of Black Lion Specialized Hospital between January and December 2020.

##### 2.3.1.2 Controls

‐ In this study, the control population consisted of children seen at the pediatric orthopedic clinic from January 2020 to December 2020 who did not have congenital clubfoot. Eligible controls were children up to 5 years of age presenting with conditions other than congenital clubfoot, including injuries.

#### 2.3.2 Exclusion Criteria

##### 2.3.2.1 Cases

Charts with incomplete data and those lost from the record office during data collection were excluded from the study. Additionally, cases involving relapsing congenital clubfoot and individuals over 5 years old were not included in the analysis.

##### 2.3.2.2 Control

Controls with clinical sign or symptom suggestive of congenital club foot were excluded. Additionally, individuals with other orthopedic conditions, such as limb deformities, skeletal dysplasia, and hip dysplasia, traumatic injuries, including fractures and sprains, and musculoskeletal disorder, such as juvenile idiopathic arthritis and muscular dystrophies, developmental conditions were also excluded.

### 2.4 Sample Size Determination and Sampling Technique

#### 2.4.1 Sample Size Determination

The primary outcome of this study was the association between potential risk factors including maternal, familial, perinatal, and environmental factors and the occurrence of congenital clubfoot in children under five. Secondary outcomes included the assessment of other potential risk factors individually. The sample size was calculated using Epi Info 7 statistical software based on the double population formula: *n* = (r + 1)/r × p(1−p) × (Zβ− Zα/2)²/(P1−P2)².

Assumptions included a 5% significance level (α), 80% power (1−β), a 95% confidence level, and a control‐to‐case ratio of 2:1. The largest estimated sample size was selected to ensure sufficient power for detecting associations with the primary outcome. Considering a 10% non‐response rate, the final sample size was 288 participants (96 cases and 192 controls) (Table 1). For practical reasons, the same sample size was applied when analyzing secondary outcomes, although the study was primarily powered to detect differences for the primary outcome.

**Table 1:** Sample size determination of case control (for objective two) was as follow.

Power	Level of significance	Proportion of exposure among control	Proportion of exposure among case	Non‐response rate	Minimum sample size
80%	5%	0.9%	7.2%	10%	288 (96 cases 192 control)

#### 2.4.2 Sampling Procedure

Medical record numbers (MRNs) of all pediatric patients were retrieved from the hospital's Pediatric Orthopedic Clinic health management information system (HMIS) registration book during the study period. The MRNs were assigned consecutive numbers based on their registration order in the HMIS. A random sampling method was then used to select patient charts from the list of MRNs, which served as the sampling frame.

### 2.5 Study Variables

#### 2.5.1 Dependent Variable

Congenital club foot.

#### 2.5.2 Independent Variables

Maternal education status, maternal age at pregnancy, maternal marital status. Maternal reproductive factors like parity, ANC visit: maternal life style, including alcohol, smoking cigarate, smoking exposure, family history congenital club foots, maternal drug use (psychiatric drug), maternal chronic illness (Diabetic mellitus, hypertension), patient characteristics like sex of the patient and parity.

### 2.6 Data Collection Tool and Procedures

Data was gathered using a structured checklist adapted from existing literature. The Health Management Information System (HMIS) registration book was consulted to retrieve the Medical Record Numbers (MRNs), which were used to locate the main patient files in the chart room. Relevant information, such as age, sex, and other key variables, was extracted from the patient cards to ensure completeness. Missing critical data, such as family history of clubfoot, was obtained through phone communication with the families. A specialized data card was used to record variables according to the study's inclusion and exclusion criteria. The data collection was carried out by four BSc nurses, with two senior BSc nurses supervising the process. The Principal Investigator (PI) provided ongoing oversight throughout the data collection period.

### 2.7 Data Quality Control

Data collectors and supervisors received 2 days of training on data collection methods, materials, and the research objectives. Daily supervision was conducted to ensure data completeness and consistency, with both supervisors overseeing the process to maintain data quality. A pretest was conducted on 5% of the total sample size of patient cards before the actual data collection, and necessary corrections were made. To assess the face validity of the questionnaire, feedback and comments were obtained from advisors and experts in the field of orthopedics.

### 2.8 Data Analysis and Interpretation

The data were reviewed for completeness after each collection session and then entered into SPSS Version 25 for analysis. Descriptive statistics, including frequencies and percentages, were calculated and presented in tables. Logistic regression analysis was conducted to identify factors associated with clubfoot. Covariates for the multiple logistic regression model were selected based on existing literature, biological plausibility, and clinical relevance, rather than solely relying on statistical significance. Multivariable logistic regression was used to calculate adjusted odds ratios (AORs) and their corresponding confidence intervals (CIs) to evaluate the strength of associations between dependent and predictor variables, with a significance level set at *p* ≤ 0.05. Model fit was assessed using the Hosmer‐Lemeshow goodness‐of‐fit test. Additionally, Variables with a VIF exceeding 10% were considered for collinearity. If high collinearity was detected, we planned to address it by either removing one of the correlated variables based on theoretical importance or prior evidence, or by combining correlated variables into a composite or categorical variable where appropriate.

## 3 Results

### 3.1 Socio Demographic Characteristics of Mother and Clubfoot Patient

A total of 286 (96 cases and 192 controls) were included to this study, with 98.6% response rate of control group; the remained 1.4% was non‐respondents due to failure to get complete chart. The mean ( ± SD) age of mother, born clubfoot cases and controls was 26.51 ( ± 4.4) and 27.42 ( ± 4.13) respectively. Almost two‐third of the mother, 56 (60.4%) of cases and 121 (64.4%) of controls were found in the age group of 25–34 years, while 84 (87.5%) of cases and 141 (75%) of controls were came from Addis Ababa. Likewise 91 (94.8%) of the cases and 177 (94.1%) of the controls were married. Regrading to the occupation, 51 (53.1%) cases and 92 (48.9%) controls were housewives. Concerning educational status, only 38(39.6%) of mother from the cases attended their secondary school and 95(50.5%) of controls had attended above secondary. This study also revealed that nearly three‐fourth clubfoot 71(74%) of cases and 113(60.1%) of controls were males (Table 2).

**Table 2:** Socio‐demographic characteristics of mothers attending Black lion specialized hospitals Ethiopia, 2021 (*n* = 284).

Variable	Categories	Case ( = 96) Frequency (%)	Control (*n* = 188) Frequency (%)
Age of the mother(year)	20–24	34 (35.4)	53 (28.2)
	25–35	58 (60.4)	121 (64.4)
	> 35	4 (4.2)	14 (7.4)
Occupation of the mother	House wife	51 (53.1)	92 (48.9)
	Student	6 (6.3)	14 (7.4)
	Government employ	13 (13.5)	41 (21.8)
	Daily Labor	13 (13.5)	19 (10.1)
	Self‐employ	12 (12.5)	22 (11.7)
	No Job	1 (1)	
Region of the mother	Addis Ababa	84 (87.5)	141 (75)
	Oromia	7 (7.3)	27 (14.4)
	Amhara	4 (4.2)	14 (7.4)
	Other	1 (1)	6 (3.2)
Marital status of the mother	Single		7 (3.7)
	Married	91 (94.8)	177 (94.1)
	Divorced	4 (4.2)	2 (1.1)
	Widowed	1 (1)	2 (1.1)
Educational status of the mother	No Education	8 (8.3)	3 (1.6)
	Primary Education	15 (15.6)	20 (10.6)
	Secondary Education	38 (39.6)	88 (46.8)
	Above Secondary	35 (36.5)	77 (41)
Sex of the child	Male	72 (75)	112 (59.6)
	Female	25 (25)	75 (39.4)

### 3.2 Maternal Obstetrics and Foot Deformity History

The study showed that more than two‐third mother, 64 (66.7%) of the cases and three‐fourth 146 (77.7%) of the controls were multipara and almost all, 93 (96.7%) of cases and 183 (97.3%) of controls had ANC visits during pregnancy. Around 94.8% (91) of cases and 178 (94.7%) of controls had planned pregnancy. This study revealed that 10 (10.4%) of the cases and 1 (0.5%) of the controls had family history of clubfoot. Within the study population, 3 cases (3.1%) and 8 controls (4.3%) reported a family history of foot deformities other than congenital clubfoot, including acquired deformities such as trauma‐related conditions (Table 3).

**Table 3:** Maternal obstetrics and foot deformity history attending Black lion specialized hospitals Ethiopia, 2021 (*n* = 284).

Variable	Categories	Cases (*n* = 96)Frequency (%)	Controls (*n* = 188)Frequency (%)
Family History Club foot	Yes	12 (12.5%)	3 (1.6%)
	No	86 (87.5%)	187 (98.4%)
Parity of the mother	Null parity	34 (35.4%)	40 (21.3%)
	Multi parity	62 (64.6%)	148 (78.7%)
Family history of Foot deformity*	Yes	3 (3.1%)	8 (4.3%)
	No	93 (96.9%)	180 (95.7%)
Planned pregnancy	Yes	91 (94.8%)	178 (94.7%)
	No	5 (5.2%)	10 (5.3%)
ANC follow up	Yes	92 (95.8%)	184 (97.9%)
	No	4 (4.2%)	4 (2.1%)

* Family history of foot deformity refers to any structural foot abnormality reported among biological relatives, excluding congenital clubfoot, and may include trauma‐related or other acquired deformities.

### 3.3 Maternal Medical, Environmental and Drug History

Regarding to alcohol drinking, 27 (28.1%) cases and 11(5.9%) controls had alcohol drinking history during pregnancy. Only one (1%) case had cigarette smoking and two (1.1%) controls were smokers. Likewise one (1%) of the cases and the controls had a history of both smoking and family history of clubfoot during pregnancy. Nearly one third, 31 (32.3%) of the cases and 17 (9%) of the controls reported that they had a history of smoking exposure during the pregnancy. The study indicated that 13(13.5%) of the cases and 14 (7.5%) of the controls had chronic illness. Regarding radiation exposure of mother, 2 (2.1%) of cases and 1 (0.5%) of controls had radiation exposure during pregnancy. Concerning to drug intake 9 (9.4%) of cases had drug intake history at the time of pregnancy and no history drug intake for controls (Table 4).

**Table 4:** ‐ Maternal medical, environmental and drug history of pregnant mother attending Black lion specialized Hospital, Ethiopia, 2021 (*n* = 284).

Variable	Categories	Cases Frequency (%)	Controls Frequency (%)
Alcohol	Yes	27 (28.1%)	11 (5.9%)
	No	69 (71.9%)	177 (94.1%)
Smoking exposure	Yes	31 (32.3%)	17 (9%)
	No	65 (67.7%)	171 (91%)
Smoking	Yes	1 (1%)	2 (1.1%)
	No	95 (99%)	186 (98.9%)
Radiation exposure	Yes	2 (2.1%)	1 (0.5%)
	No	94 (97.9%)	187 (99.5%)
Club foot and smoking	Yes	1 (1%)	1 (0.5%)
	No	95 (99%)	187 (99.5%)
Chronic illness	Yes	13 (13.5%)	14 (7.5%)
	No	83 (86.5%)	173 (92.5%)
Drug intake	Yes	9 (9.4%)	0 (0%)
	No	87 (90.6%)	188 (100%)

### 3.4 Determinants of Congenital Clubfoot

A multivariable logistic regression analysis was conducted to identify determinants of congenital clubfoot, based on clinical relevance and existing literature. The following variables were included in the final model: age, educational level, sex of children, family history of clubfoot, alcohol consumption history, smoking exposure, and chronic illness, as they were identified as significant or clinically relevant from prior studies and expert opinion. Adjusted odds ratios (AORs) with corresponding 95% confidence intervals (CIs) were used to assess the strength of the associations.

The variables were entered into a multivariable logistic regression model using the backward stepwise approach, with adjustments made for covariates. The model was considered a good fit as there was no multicollinearity between the variables, and the Hosmer‐Lemeshow test yielded a *p*‐value of 0.503. Influential observations were assessed using standardized residuals and Cook's distance. Sensitivity analysis excluding influential cases showed that the results remained consistent, supporting the robustness of the logistic regression model.

In this study, no formal maternal education was significantly associated with clubfoot. Pregnant mothers who had no formal maternal education 8.29‐fold (AOR = 8.29, 95% CI: 1.77–38.84) higher odds of developing clubfoot compared to the odds of mothers who had formal education diploma & above. Male was identified as one of the determinants of congenital clubfoot. The odds of developing clubfoot among born children who had male sex were 2.08 times (AOR = 2.08, 95% CI: 1.10–3.92) higher than compared to female.

Study participants who had maternal smoking exposure during this pregnancy showed a significant association with clubfoot. The odds of developing clubfoot were 4.83 times (AOR = 4.83, 95% CI: 2.30–10.14) higher among those who had smoking exposure compared to their counterparts. Similarly, alcohol drinking was found to be a determinant of clubfoot. Pregnant women who had drink alcohol during pregnancy were 5.39 times (AOR = 5.60, 95% CI: 2.31–12.56) more likely to develop clubfoot compared with those who had no history of alcohol drinking.

The current study found that maternal chronic illness was significantly associated with clubfoot. Pregnant mothers who had chronic illness 2.57‐fold (AOR = 2.57, 95% CI: 1.04–6.33) higher odds of developing clubfoot compared to the odds of mothers who hadn't chronic illness. The study also indicated that family history of clubfoot was also identified as a determinant of clubfoot. The odds ratio of developing clubfoot were 12.78 times (AOR = 12.8, 95% CI: 3.17–51.60) higher for clubfoot with family history of the born children compared with born children with no with family history of clubfoot (Table 5).

**Table 5:** Bi‐variable and multi‐variable logistic regression analysis for determinants of clubfoot among mother & children attending Black lion Specialized Hospital, Ethiopia, 2021 (*n* = 284).

Variables	Category	Clubfoot status		COR(95%CI)	AOR (95%CI)	*p*
		Cases N (%)	Control N (%)			
Age of mother	20–24	34 (35.4)	53 (28.2)	2.25 (0.68–7.39)	2.35 (0.59–9.32)	0.22
	25–34	58 (60.4)	121 (64.4)	1.68 (0.53–5.32)	2.01 (0.52–7.75)	0.31
	> 35	4 (4.2)	14 (7.4)	1	1	
Educational level	No education	8 (8.3)	3 (1.6)	5.87 (1.47–23.45)	8.29 (1.77–38.84)	0.007
	Primary	15 (15.6)	20 (10.6)	1.65 (0.76–3.60)	2.47 (0.97–6.28)	0.054
	Secondary	38 (39.6)	88 (46.8)	0.79(0.55–1.65)	1.09 (0.58–2.07)	0.79
	Above secondary	35 (36.5)	77 (41)	1	1	
Sex	Male	72 (75)	112 (59.6)	1.86 (1.1–3.24)	2.08 (1.10–3.92)	0.023
	Female	25 (25)	75 (39.4)	1	1	
Smoking exposure	Yes	31 (32.3%)	17 (9%)	4.80 (2.45–9.25)	4.83 (2.30–10.14)	< 0.001
	No	65 (67.7%)	171 (91%)	1	1	
Alcohol drinking	Yes	27 (28.1%)	11 (5.9%)	6.30 (2.96–13.39)	5.39 (2.31–12.56)	< 0.001
	No	69 (71.9%)	177 (94.1%)	1	1	
Chronic illness	Yes	13 (13.5%)	14 (7.5%)	2.57 (1.04–6.33)	2.32 (0.96–5.58)	0.04
	No	83 (86.5%)	173 (92.5%)	1	1	
Family history of clubfoot	Yes	12 (12.5%)	3 (1.6%)	8.81 (2.42–32.04)	12.78 (3.17–51.60)	< 0.001
	No	86 (87.5%)	187 (98.4%)	1	1	

## 4 Discussion

In low‐income countries, limited access to healthcare services poses significant challenges for early diagnosis and treatment of clubfoot, leading to a high burden of disease and delayed intervention [23]. With healthcare service limited and the costs being a significant barrier, prevention strategies focusing on early detection, education, and access to treatment are crucial for mitigating the impact of clubfoot in Ethiopia. In this study, factors showed significant association with clubfoot including, no formal educational, being male patient, family history of club foot, maternal smoking exposure, chronic illness and maternal alcoholic, show significant association with congenital club foot. These findings emphasize the need for targeted interventions focusing on maternal health education, lifestyle modifications, and early screening programs to reduce the risk of congenital clubfoot. Such interventions may help reduce exposure to modifiable maternal risk factors identified in this study, particularly alcohol consumption, passive smoking, and unmanaged chronic illnesses during pregnancy.

Early detection of congenital clubfoot through routine neonatal examinations and strengthened antenatal care services may facilitate timely referral to specialized orthopedic clinics, enabling early initiation of the Ponseti method, which has been shown to be highly effective when started in early infancy [24, 25]. In addition, maternal health education provided during antenatal care visits could play an important role in reducing exposure to modifiable risk factors identified in this study, such as alcohol consumption and passive smoking [26, 27, 28]. Educational interventions may also improve maternal awareness regarding the importance of healthy lifestyle practices during pregnancy. Furthermore, improving access to appropriate orthopedic treatment and strengthening referral systems may reduce delays in care and prevent long‐term disability associated with untreated clubfoot [23]. Beyond individual awareness, low maternal education often reflects broader socio‐economic disadvantages, including limited access to nutrition, healthcare services, and safe living environments, which may also contribute to the risk of clubfoot. Addressing these systemic factors alongside targeted education may further reduce the burden of congenital clubfoot.

In the present study the odd of club foot occurrence was higher for mother that had no formal educational compared to diploma and above level. This finding is consistence with other studies conducted USA and Peru [16, 29]. This could be explained by the fact that in Ethiopia, women's higher education enrollment rate is disproportionately low, and only 4% have completed beyond secondary [30]. Therefore, women with no formal education may be less aware of environmental factors that contribute to clubfoot. Beyond limited awareness of known risk factors, low maternal education may reflect broader socioeconomic disadvantages, including reduced access to healthcare services, inadequate nutrition, and suboptimal living environments, which could contribute to the occurrence of congenital clubfoot through additional, potentially unknown mechanisms. Furthermore, intergenerational poverty may exacerbate these effects by limiting access to healthcare, early screening, and adherence to effective treatment protocols, as reported by Pigeolet et al. [23], highlighting the cross‐cutting impact of socioeconomic disadvantage on both the development and management of clubfoot. Enhancing awareness of potential environmental risk factors during antenatal care can help identify potential issues early. Avoiding exposure to known teratogens, such as certain medications, alcohol, and tobacco during pregnancy may help reduce the risk of birth defects, including clubfoot.

This study also revealed that the odds of club foot were 2 times higher in males compared to females. The results of this study was in line with studies conducted in Peru [16], USA [29], Romania [31], Sicily [32], & China [33]. The possible justification for the association of males with club foot might be the fact that, in order to inherit club foot, female need to have a great number of susceptibility genes(carter effect) than males [34]

Patients who had family history of clubfoot for this study were likely to develop clubfoot compared to those who had no family history of clubfoot. This finding was supported by previous data conducting in Atlanta [35], the New Zealand Polynesian population [19], Sicily [32], Norway [14], & Multi state of USA [29]. The explanation for this association probably could be complex inheritance factors of multiple defected genes through family [34], like clubfoot is 33% for monozygotic twins versus only 3% for dizygotic twins [36]. As a result, if there is a family history of clubfoot or other congenital anomalies, genetic counseling may be beneficial. This can help assess the risk of recurrence and provide information about potential genetic factors involved.

The present study also revealed that chronic illness was identified as a determinant of clubfoot. This finding is in agreement with studies conducted in USA [15, 29]. While some factors contributing to clubfoot, such as chronic illnesses, may not be entirely preventable, maintaining good overall health and effectively managing chronic conditions during pregnancy can support the best possible outcomes for both the mother and her baby. It is important to recognize that socio‐economic determinants and access to healthcare also play a significant role in managing these conditions [37]. Beyond individual behaviors, the development of congenital clubfoot is also influenced by broader structural and socio‐economic determinants, including poverty, intergenerational disadvantage, limited access to healthcare, inadequate maternal nutrition, and commercial determinants of health such as exposure to alcohol and tobacco [1, 23]. These structural conditions may limit the ability of pregnant women to effectively manage health risks during pregnancy. While several risk factors for clubfoot have been reported in previous studies, much of the available evidence originates from high‐income settings. In contrast, evidence from low‐income countries, particularly in sub‐Saharan Africa, remains limited. The present study contributes context‐specific evidence from Ethiopia by examining maternal education, lifestyle exposures, chronic illness, and family history within a resource‐constrained health system. By identifying how these factors interact within this setting, the study helps highlight locally relevant opportunities for prevention, including strengthening antenatal care services, maternal health education, lifestyle counseling, and early neonatal screening to facilitate timely referral for the Ponseti method. Furthermore, addressing broader socio‐economic barriers and improving access to maternal and child health services may help mitigate modifiable risk exposures during pregnancy.

Maternal smoking exposure has also been identified as risk factor for developing clubfoot by four times than of non‐exposure. This study produce similar finding with the study conducted in, USA [35], & Peru [16]. The justification for this may be, nearly one‐third of adult Ethiopians are exposed to secondhand smoke at work or home [38], this exposure leads to the compromise effect of smoking on placenta and fetal vasculature by nicotine [39] might be contributing to occurrence of congenital clubfoot. Because of this, counseling to the mother about the various negative impact associated with smoking during pregnancy is essential, including the increased risk of birth defects like clubfoot by emphasize that exposure to cigarette smoke can have detrimental effects on fetal development.

In the present study further showed, the odd of club foot was higher in maternal alcoholic compared to non‐alcoholic mother. Similarly, a study conducted in New York, North Carolina [40, 41] maternal alcohol history were showed a positive association with club foot. It might be also due to that alcohol induce reactive oxygen species, decreased endogenous antioxidant levels, mitochondrial damage, placental vasoconstriction, and inhibition of cofactors required for fetal growth and development [42]. Because of this, it is important to increase awareness regarding the negative aspects of alcohol drinking during pregnancy, by devise the mother to abstain from alcohol entirely during pregnancy and inform them about no known safe amount of alcohol consumption during pregnancy.

Although most western studies had reported a significant association between maternal smoking and club foot [14, 15, 35, 40, 43, 44], in our study, significant association was not found between maternal smoking and club foot, most probably due to, in Ethiopia, tobacco use was strongly sex linked, among reproductive‐age women, prevalence of tobacco use was 0.8% women versus 6.2% of men [45]. In Oceania, Asia, Europe, & America reproductive‐age women smokers rate were, 36%, 14%, 38% and 31% respectively [46].

Finally, we recommend that future studies include larger, multi‐center populations to enhance the robustness and generalizability of findings. Exploration of genetic contributions, environmental exposures, and geographical factors is encouraged to better understand congenital clubfoot determinants. Additionally, integrating qualitative approaches with quantitative analyses could inform more culturally appropriate and practical maternal health interventions. Finally, future research should investigate potential teratogenic or toxic drug effects on congenital clubfoot. To further strengthen the generalizability and clinical relevance of future work, studies should be designed as prospective, multi‐center investigations across different regions of Ethiopia. Standardized data collection protocols should be implemented to systematically capture maternal, familial, and environmental risk factors. Maternal chronic illnesses should be categorized into specific subtypes (diabetes mellitus, hypertension), and detailed birth histories should be obtained. Such a framework would allow for more robust analyses and provide findings that are representative of the broader Ethiopian population.

### 4.1 Strength and Limitations of the Study

An important strength of the study is the effort made to obtain crucial data that was missing from patient charts. To address this gap, we conducted direct phone communications with families to gather this information, ensuring a more comprehensive and accurate dataset. This proactive approach helped mitigate the impact of incomplete documentation and enhanced the reliability of the study's findings. However this study was done in one hospital and data were collected over 1 year retrospectively, so the findings may fail to reflect the true picture of club foot risk factors. Additionally, although phone communications were conducted to obtain maternal information, the details regarding maternal chronic illnesses were not sufficiently detailed to categorize them into specific subtypes (diabetes mellitus, hypertension). This represents a limitation of the study and should be addressed in future prospective multi‐center research.

## 5 Conclusion

This research identified several factors contributing to congenital clubfoot, including low maternal education, being male, family history of clubfoot, exposure to smoking, chronic illnesses, and alcohol use. Although maternal smoking did not emerge as a significant factor in this study, it emphasizes the need for comprehensive antenatal care to educate mothers about various risks, including smoking. The study advocates for increased awareness and nutritional support during pregnancy and encourages empowering pregnant women to recognize and manage potential determinants of clubfoot. Additionally, the research highlights the need for further, extensive investigations by other researchers to confirm and expand upon these findings.

## Author Contributions

**Seid Mohammed Abdu:** conceptualization, data curation, investigation, formal analysis, methodology, writing – review and editing, writing – original draft, software. **Birhanu Ayana:** data curation, software, formal analysis, writing – original draft, writing – review and editing. **Girma Seyoum:** writing – review and editing, writing – original draft, data curation, formal analysis, software, methodology, investigation.

## Funding

The authors have nothing to report.

## Ethics Statement

The study was conducted following the principles of the Declaration of Helsinki. Ethical approval was obtained from the Departmental Research Ethical Review Committee of Addis Ababa University in the Anatomy Department (DRERC) (Meeting No.: DRERC/09/21). Additionally, an informed consent waiver was provided by the same committee due to the retrospective nature of the study, which made it impractical to obtain consent from all patients’ guardians. The ethical clearance and cooperation letter was sent to the Black Lion Specialized Hospital Outpatient Department Directorate to obtain permission to collect the data. The confidentiality of patient information was maintained throughout the process by collecting data anonymously. After data collection, the raw data was secured and was not accessed by anyone except the principal investigator.

## Consent

The authors have nothing to disclose.

## Conflicts of Interest

The authors declare no conflicts of interest.

## 1 Transparency Statement

The lead author Seid Mohammed Abdu, affirms that this manuscript is an honest, accurate, and transparent account of the study being reported; that no important aspects of the study have been omitted; and that any discrepancies from the study as planned (and, if relevant, registered) have been explained.

## Data Availability

Required data will be available upon request of the corresponding author.
